# Prediabetes and diabetes were attributed to the prevalence and severity of sarcopenia in middle-aged and elderly adults

**DOI:** 10.1186/s13098-024-01355-3

**Published:** 2024-06-02

**Authors:** Jing Yuan, Pu Jia

**Affiliations:** 1 Department of Endocrinology, Beijing Tongren Hospital, Capital Medical University, Beijing, 100730 China; 2 Department of Orthopedics, Beijing Friendship Hospital, Capital Medical University, Beijing, 100050 China

**Keywords:** Sarcopenia, Prediabetes, Diabetes, Hyperglycemia

## Abstract

**Background:**

Sarcopenia and diabetes are both prevalent health problems worldwide. However, little is known about the relationship between prediabetes and the prevalence and severity of sarcopenia. Therefore, the current study aimed to explore the association between glucose status and the components of sarcopenia, including low muscle mass (LMM), low muscle strength (LMS) and low gait speed (LGS) in US adults.

**Methods:**

Data from the 1999 to 2002 National Health and Nutrition Examination Survey (NHANES) were analyzed. A total of 4002 participants aged ≥ 50 years with available information on glucose status (NGR: 1939 cases; prediabetes: 1172 cases; diabetes: 891 cases) and sarcopenia were included in this study. Sarcopenia was defined according to the Foundation for National Institute of Health criteria. Muscle mass, muscle strength and gait speed were used to evaluate sarcopenia and its severity. Weighed multivariable logistic regression were used to explore the association between glucose status and the components of sarcopenia. The hypothetical population attributable fraction (PAF) for the glucose status was also calculated.

**Results:**

The mean age of the cohort was 63.01 ± 9.89 years, with 49.4% being male. Multiple logistic regression analysis suggested that diabetes was an independent risk factor for sarcopenia (OR = 5.470, 95% CI 1.551–19.296) and showed a marginal association with severe sarcopenia (OR = 10.693, 95% CI 0.955–119.73) compared to NGR in men, but not in women. Additionally, prediabetes was independently associated with severe sarcopenia (OR = 3.647, 95% CI 1.532–8.697), LMS (OR = 1.472, 95% CI 1.018–2.127) and LGS (OR = 1.673, 95% CI 1.054–2.655) in the entire cohort. When stratifying by gender, we further observed that prediabetes was significantly associated with LMS in men (OR = 1.897, 95% CI 1.019–3.543) and related to LMM (OR = 3.174, 95% CI 1.287–7.829) and LGS (OR = 2.075, 95% CI 1.155–3.727) in women. HbA1c was positively associated with the prevalence of sarcopenia in men (OR = 1.993, 95% CI 1.511–2.629). PAF showed that diabetes accounted for 16.3% of observed sarcopenia cases. Maintaining NGR in the entire population could have prevented 38.5% of sarcopenia cases and 50.9% of severe sarcopenia cases.

**Conclusions:**

Prediabetes and diabetes were independently associated with the prevalence and severity of sarcopenia in US population. Slowing down the progression of hyperglycemia could have prevented a significant proportion of sarcopenia cases.

**Supplementary Information:**

The online version contains supplementary material available at 10.1186/s13098-024-01355-3.

## Introduction

Sarcopenia is a progressive skeletal muscle disorder characterized by the degenerative loss of muscle mass (LMM) and impaired muscle strength. It is associated with various adverse health outcomes, including increased risk of falls and fractures [1, 2], disability of physical performance [3], frailty and mortality [4]. Although certain risk factors such as older age, reduced physical activities and lower body weight [5] have been identified, the pathophysiology of sarcopenia remains incompletely understood. As a result, there is growing interest in identifying additional risk factors and developing effective preventive strategies.

Currently, the development of sarcopenia in diabetic patients has been paid great attention. A previous meta-analysis reported an increased risk of physical disability in individuals with diabetes [6]. Furthermore, several studies suggested that sarcopenia, defined by lower muscle strength (LMS) and/or muscle mass, was more prevalent in diabetic patients than in non-diabetic individuals [7–9]. Underlying mechanisms for this association include chronic low-grade inflammation [10] and increased insulin resistance [11]. Therefore, diabetes is recognized to be a potential risk factor for sarcopenia. However, it remains unclear whether diabetes is related to the severity of sarcopenia.

Prediabetes serves as an intermediate stage between normo-glycaemia and diabetes, with an annualized conversion rate of 5–10% to diabetes [12]. To our knowledge, there were only a few studies focusing on the relationship between prediabetes and sarcopenia [13, 14]. A study from Japan confirmed that prediabetes was linked to sarcopenia in men, but not in women [14]. Previous analysis utilizing data from National Health and Nutrition Examination Survey (NHANES) [15] evaluated the relationship between prediabetes and sarcopenia. In this study, sarcopenia was defined solely based on low muscle mass, without considering measures of muscle strength and walking speed, which reflect the quality of appendicular skeletal muscle mass (ASM). As a result, the available evidence on this topic remains inconclusive, highlighting the need for further investigation.

In the current study, using the nationally representative NHANES data from 1999 to 2002, we aimed to (1) evaluate the prevalence and severity of sarcopenia based on LMS, LMM and low gait speed (LGS) in patients with prediabetes and diabetes; (2) investigate whether prediabetes and diabetes were independently associated with sarcopenia; (3) quantify the number of sarcopenia cases that would be prevented if the entire population maintained normal glucose regulation (NGR).

## Methods

### Study design

NHANES is a major and continuous ongoing program of National Center for Health Statistics (NCHS), which is a part of the Centers for Disease Control and Prevention (CDC). The survey aims to monitor a variety of health and nutritional status of adults and children in the US, data of which usually used in epidemiological studies and health sciences research. To represent the US population of all ages comprehensively and yield reliable statistics, the survey was designed by a multistage, complex stratified probability sampling. The data of NHANES 1999–2002 were included in this study due to the availability of measurements for muscle mass, muscle strength and gait speed during these survey cycles. The NHANES study protocol were approved by the NCHS Ethics Review Board, and written informed consent was obtained from all participants.

### Study population

We restricted our cohort to subjects aged 50 years and older. Among the 21,004 individuals in the NHANES study from 1999 to 2002, 4983 cases were aged ≥ 50 years. According to the inclusion criterion, individuals with missing data of glucose status, muscle mass, knee extensor strength and gait speed were excluded from the study. Ultimately, there were 1939 subjects with NGR, 1172 with prediabetes and 891 with diabetes who had valid data of components of sarcopenia.

### Measurements

Standard questionnaires were used to record information via household interviews about age, gender, race/ethnicity, educational levels, ratio of family income to poverty (PIR) and marital status. Race/ethnicity was categorized into non-Hispanic white, non-Hispanic black, Mexican American and other races. Educational levels were categorized as under high school, high school or equivalent, above high school. PIR was classified as ≤ 1, 1 < to ≤ 3, > 3, with lower value representing higher level of poverty. Marital status was categorized into three groups: married/living with partner, widowed/divorced/separated, and never married. Data of smoked at least 100 cigarettes in life (yes/no), vigorous activities (yes/no) and sedentary activities (≥ 3 h/day; 1–3 h/day; ≤ 1 h/day), history of cancer (yes/no) or osteoporosis/brittle bones (yes/no) were also collected through the NHANES database. Physical examinations were conducted at the NHANES mobile examination center (MEC). Body mass index (BMI) was calculated by dividing weight (kg) by the square of height (m^2^). Waist circumference were measured using a tape in a horizontal plane at the position of just above the uppermost lateral border of the ilium. Systolic blood pressure (SBP) and diastolic blood pressure (DBP) were obtained with a mercury sphygmomanometer after resting in a seated position for 5 min.

Blood specimens were collected at a 9-h overnight fast status, and stored under appropriate refrigerated or frozen conditions for transport to laboratories for analysis. Glycohemoglobin (HbA1c) was tested in whole blood specimen by the method of high-performance liquid chromatography. Fasting plasma glucose (FPG) were measured by enzymatic method. Serum insulin (INS) were examined by a two-site enzyme immunoassay performing on Tosoh AIA System analyzer. Homeostasis model assessment insulin resistance (HOMA-IR) was calculated by the following equation: (fasting INS × FPG)/22.5. Serum 25-hydroxyvitamin D (25(OH)D) were measured by a radioimmunoassay (DiaSorin RIA kit) method. Serum total bilirubin (TBL), creatinine (sCr), uric acid (UA) and total cholesterol (TC) were tested with automatic biochemistry analyzer (Beckman, US). All methods were performed in accordance with the relevant guidelines and regulations. More details of the procedures were at the NHANES website.

### Definition of glucose status

According to the American Diabetes Association (ADA) criteria [16], the diagnosis of diabetes and prediabetes was determined. Diabetes was defined based on at least one of following conditions: (1) participants responded “yes” to the question “Have you ever been told by a doctor that you have diabetes?”; (2) FPG ≥ 7.0 mmol/L; (3) HbA1c ≥ 6.5%. Prediabetes was defined by a positive answer to the question “Have you ever been told by a doctor that you have prediabetes?” or had FPG ≥ 6.1, < 7.0 mmol/L, or HbA1c ≥ 5.7%, < 6.5%.

### The measurement of components to define Sarcopenia

Dual energy x-ray absorptiometry (DXA) scans of whole body were conducted using a Hologic QDR-4500A fan-beam densitometer (Hologic, Inc., Bedford, Massachusetts). Hologic software version was utilized to administer all scans. Individuals with weight over 136.4 kg or height over 192.5 cm were excluded due to the limitation of the DXA table. Additionally, participants who had used radiographic contrast material (barium) within the past 7 days were ineligible for the DXA scan. The DXA examinations were conducted by trained and certified radiology technologists, and quality control were maintained in the process of DXA scan and data collection, including adherence to a rigorous phantom scanning schedule.

Fat mass and lean mass of the arms, legs, trunk and total body were assessed by DXA. Appendicular lean muscle mass (ALM) was defined as the sum of fat-free muscle mass in the four extremities, while appendicular fat mass (AFM) was defined as the sum of fat mass in the four extremities. Appendicular fat-muscle ratio (AFMR) was calculated as ALM/AFM. To evaluate muscle mass status, ALM adjusted for BMI (ALM/BMI) and ALM adjusted for weight (ALM/weight) were used. In our study, LMM was defined according to the foundation for the national institutes of health (FNIH) sarcopenia project, with ALM/BMI cutoff values < 0.789 for men and < 0.512 for women [17]. A 6-m walk test were timed in the MEC using a hand-held stopwatch. LGS was defined as a speed < 0.8 m/s based on the FNIH Sarcopenia Project. Knee extensor strength was measured using a Kin Com dynamometer, and as suggested by a previous study [18], participants with knee extensor strength < 262.25 N for male and < 215.10 N for female were diagnosed as having LMS. Participants with LMS and LMM were diagnosed with sarcopenia, and sarcopenia patients with LGS were considered to have severe sarcopenia.

### Statistical analysis

According to the NHANES Analytic Guidelines, weighting represents several features of the survey: (1) the differential probabilities of selection for the sampling domains; (2) survey nonresponse; (3) differences between the final sample distribution and the target population distribution. Sample weights, strata, primary sampling unit and cluster were employed to interpret the complicate NHANES survey design.

Continuous variables were expressed as weighted mean ± standard deviation, while categorical measures were reported as weighted percentage. One-way analysis of variance (ANOVA) were used to compare means and chi-squared test were utilized for percentage comparisons. The prevalence of sarcopenia and its components were assessed in the entire cohort and in subgroups stratified by gender and age. Age categories were classified as: 50–59 years, 60–69 years, 70–79 years and ≥ 80 years. Furthermore, we evaluated whether glucose status was associated with sarcopenia, severe sarcopenia, LMS, LMM and LGS by multivariable logistic regression analysis [odds ratios (ORs) and 95% confidence interval (CI)] in three models. Model 1 was an unadjusted model. Model 2 included adjustment for age, gender, race and BMI. Model 3 included adjustments from model 2 as well as waist circumference, percentage of total fat, education level, marital status, PIR, smoking history, vigorous activities, sedentary activities, serum 25(OH)D, TBL, sCr, UA, TC and history of cancer, osteoporosis or brittle bones.

We also calculated population of attributable fraction (PAF) to estimate the percentage of sarcopenia cases that could potentially be prevented under two hypothetical scenarios: (1) “partial prevention”, indicates the proportion of sarcopenia attributable to having diabetes (diabetes vs. NGR and prediabetes); and (2) “comprehensive prevention”, defined as the scenario in which the entire population maintained NGR status (diabetes and prediabetes vs. NGR). PAFs were calculated using the following equation: *PAF* = ∑_*i*_* pd*_*i*_(*RR*_*i*_ − 1/*RR*_*i*_), where *pd*_*i*_ is the proportion of total incidence cases observed in the *i*th glucose status category and *RR*_*i*_ is the adjusted rate ratio for the *i*th exposure category.

All analysis were performed with STATA 15.0 software (StataCorp LLC, College Station, TX, USA). A two-sided *P* value < 0.05 was considered statistically significant.

## Results

### Characteristics of the participants across the two survey cycles

The weighted prevalence of prediabetes and diabetes in this cohort were 27.5% and 16.7% respectively. Overall, the mean age of the study subjects was 63.01 ± 9.89 years, with 47.4% being male. The clinical characteristics of the study participants were summarized in Table 1. Those with prediabetes and diabetes had increased age, less proportions of non-Hispanic Whites, lower education level, lower PIR, engaged in less vigorous activities, had longer duration of sedentary activities and higher level of SBP compared to those with NGR. Furthermore, significant difference were observed in history of osteoporosis/brittle bones, levels of HbA1c, FPG, serum INS, HOMA-IR, sCr, UA, TBL and 25(OH)D among the three groups.

**Table 1:** Weighted characteristics of US adults ≥ 50 years old with NGR, prediabetes and diabetes, 1999–2002

	NGR (n = 1939)	Prediabetes (n = 1172)	Diabetes (n = 891)	*P*
Age (years)	61.81 ± 9.71	64.43 ± 10.18	64.70 ± 9.49	< 0.001
Male (%)	44.3	50.5	50.0	0.019
Race/Ethnicity				
Non-Hispanic white (%)	85.1	75.1	65.3	< 0.001
Non-Hispanic black (%)	5.5	9.2	15.9	
Mexican American (%)	2.9	3.8	5.6	
Other races (%)	6.5	11.9	13.2	
Educational level				
Under high school (%)	20.4	28.4	40.9	< 0.001
High school or equivalent (%)	25.0	26.8	25.2	
Above high school (%)	54.6	44.8	33.9	
Marital status				
Married/living with partner (%)	71.3	70.0	61.4	0.015
Widowed/divorced/separated (%)	25.6	26.4	32.6	
Never married (%)	3.1	3.6	6.0	
PIR				
≤ 1 (%)	8.7	12.0	17.4	< 0.001
1 < to ≤ 3 (%)	31.0	39.6	45.1	
> 3 (%)	60.3	48.4	37.5	
Smoked ≥ 100 cigarettes in life (%)	55.0	54.8	54.9	0.993
Vigorous activities (%)	30.2	18.2	19.4	< 0.001
Sedentary activities				
< 1 h/day	11.6	11.1	8.9	< 0.001
1–3 h/day	64.3	57.5	55.2	
≥ 3 h/day	24.1	31.4	35.9	
Cancer (%)	14.3	15.4	16.6	0.442
Osteoporosis or brittle bones (%)	9.3	6.2	10.0	0.015
SBP (mmHg)	133.62 ± 21.70	138.83 ± 22.40	137.70 ± 22.31	< 0.001
DBP (mmHg)	74.21 ± 13.00	72.97 ± 15.62	69.52 ± 16.29	< 0.001
HbA1c (%)	5.27 ± 0.24	5.70 ± 0.32	7.47 ± 1.81	< 0.001
FPG (mmol/L)	4.92 ± 0.36	5.58 ± 0.57	8.44 ± 4.00	< 0.001
INS (µU/mL)	8.51 ± 4.89	12.89 ± 8.40	24.50 ± 32.60	< 0.001
HOMA-IR	2.21 ± 1.30	3.86 ± 2.65	11.27 ± 12.93	< 0.001
TBL (µmol/L)	11.58 ± 5.07	11.08 ± 4.64	10.71 ± 4.25	< 0.001
sCr (µmol/L)	75.97 ± 44.13	77.51 ± 29.91	85.66 ± 71.25	< 0.001
UA (µmol/L)	320.23 ± 86.82	352.87 ± 85.12	339.82 ± 96.13	< 0.001
TC (mmol/L)	5.46 ± 0.99	5.50 ± 1.03	5.23 ± 1.20	< 0.001
Serum 25(OH)D (nmol/L)	63.73 ± 20.56	59.71 ± 20.20	55.55 ± 18.85	< 0.001

Mean ± SD for continuous variables; proportion for categorical variables

*NGR* normal glucose regulation, *PIR* ratio of family income to poverty, *SBP* systolic blood pressure, *DBP* diastolic blood pressure, *HbA1c* hemoglobin A1c, *FPG* fasting plasma glucose, *TBL* total bilirubin, *sCr* serum creatinine, *UA* uric acid, *TC* total cholesterol, *25(OH)D* 25-hydroxyvitamin D

The components of body composition were shown in Table 2. Compared to individuals with NGR, subjects with prediabetes and diabetes tended to have increased waist circumference and BMI; ALM were relatively higher in prediabetes and diabetes, along with corresponding higher levels of AFM. However, considering higher prevalence of obesity in the prediabetes and diabetes cohorts and the impact of obesity on muscle system, BMI or weight were adjusted when evaluating the status of muscle mass. Interestingly, ALM/weight, ALM/BMI were decreased in patients with prediabetes and diabetes. The similar results were also observed when muscle mass were assessed in the arms and legs respectively. The proportion of LMS did not show statistically significant difference among the groups, while the prevalence of LMM and LGS was higher in prediabetes and diabetes. The rate of sarcopenia were 3.8% for NGR, 6.6% for prediabetes and 7.6% for diabetes (*P* = 0.003), and the prevalence of severe sarcopenia were 1.6% for NGR, 2.4% for prediabetes and 3.9% for diabetes (*P* = 0.036).

**Table 2:** The components of body composition in the cohort

	NGR (n = 1939)	Prediabetes (n = 1172)	Diabetes (n = 891)	*P*
Waist circumference (cm)	96.02 ± 13.59	102.26 ± 13.59	107.36 ± 14.45	< 0.001
BMI (kg/m^2^)	27.36 ± 5.32	29.39 ± 5.78	31.37 ± 6.61	< 0.001
ALM (kg)	20.39 ± 5.71	21.24 ± 5.74	22.22 ± 5.89	< 0.001
AFM (kg)	12.89 ± 4.99	13.58 ± 5.38	14.19 ± 6.18	< 0.001
AFMR	0.677 ± 0.294	0.674 ± 0.281	0.667 ± 0.283	0.725
ALM/weight	0.264 ± 0.042	0.259 ± 0.039	0.256 ± 0.040	< 0.001
ALM/BMI	0.753 ± 0.188	0.732 ± 0.180	0.718 ± 0.173	< 0.001
ALM (Arm) (kg)	5.43 ± 1.92	5.76 ± 1.87	6.00 ± 1.92	< 0.001
AFM (Arm) (kg)	3.34 ± 1.34	3.73 ± 1.50	4.20 ± 1.78	< 0.001
AFMR (Arm)	0.687 ± 0.339	0.706 ± 0.329	0.763 ± 0.362	< 0.001
ALM (Arm)/weight	0.070 ± 0.017	0.070 ± 0.016	0.069 ± 0.016	< 0.001
ALM (Arm)/BMI	0.201 ± 0.066	0.199 ± 0.062	0.195 ± 0.061	< 0.001
ALM (Leg) (kg)	14.96 ± 3.91	15.48 ± 3.98	16.21 ± 4.13	< 0.001
AFM (Leg) (kg)	9.55 ± 3.84	9.85 ± 4.07	9.98 ± 4.61	0.020
AFMR (Leg)	0.675 ± 0.288	0.664 ± 0.274	0.635 ± 0.271	0.006
ALM (Leg)/weight	0.194 ± 0.027	0.188 ± 0.025	0.187 ± 0.026	< 0.001
ALM (Leg)/BMI	0.552 ± 0.126	0.533 ± 0.121	0.523 ± 0.115	< 0.001
Trunk muscle (kg)	24.10 ± 5.64	25.24 ± 5.80	26.85 ± 5.95	< 0.001
Trunk fat (kg)	13.97 ± 5.43	16.19 ± 5.91	17.82 ± 6.74	< 0.001
Truck percent fat (%)	35.44 ± 8.09	37.91 ± 7.57	38.57 ± 7.65	< 0.001
Total percent fat (%)	35.67 ± 8.16	36.97 ± 7.83	37.27 ± 7.88	< 0.001
Low muscle strength (%)	21.8	25.3	24.2	0.227
Low muscle mass (%)	11.9	19.8	25.2	< 0.001
Low gait speed (%)	11.6	19.2	30.6	< 0.001
Sarcopenia (%)	3.8	6.6	7.6	0.003
Severe sarcopenia (%)	1.6	2.4	3.9	0.036

Mean ± SD for continuous variables; proportion for categorical variables

*BMI* body mass index, *ALM* appendicular lean muscle mass, *AFM* appendicular fat mass, *AFMR* appendicular fat-muscle ratio

### Sarcopenia status in sex-and-age stratification analysis

**Fig. 1:**
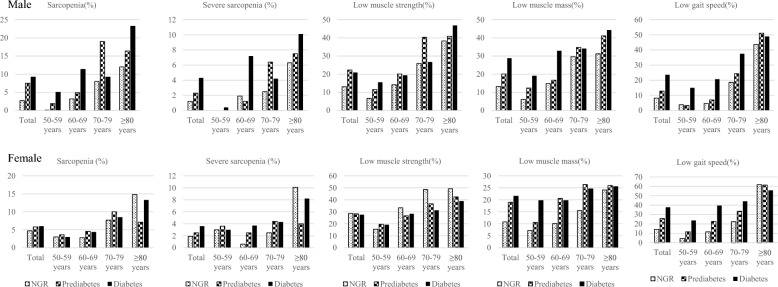
The prevalence of sarcopenia in US participants with NGR, prediabetes and diabetes stratified by age and sex

As depicted in Fig. 1 and Supplementary Table 1, the prevalence of sarcopenia among men was 2.7% for NGR, 7.5% for prediabetes and 9.2% for diabetes (*P* < 0.001), and the corresponding indicators in female were 4.7%, 5.8% and 6.0%, respectively (*P* = 0.567). Furthermore, patients with prediabetes and diabetes in men had a higher proportion of severe sarcopenia (NGR: 1.2%; prediabetes: 2.3%; diabetes 4.3%, *P* = 0.017), LMS (NGR: 13.1%; prediabetes: 22.3%; diabetes 20.9%, *P* = 0.004), LMM (NGR: 13.2%; prediabetes: 20.1%; diabetes 28.8%, *P* < 0.001) and LGS (NGR: 8.2%; prediabetes: 12.8%; diabetes 23.6%, *P* < 0.001), while the difference of the analysis in subgroup of women were only observed in LMM (NGR: 10.8%; prediabetes: 19.0%; diabetes 21.5%, *P* < 0.001) and LGS (NGR: 14.3%; prediabetes: 25.8%; diabetes 37.7%, *P* < 0.001) across the three groups. When stratifying by different age groups, we found that glucose status had a greater impact on the components of sarcopenia (LMM and LGS) in individuals aged 50–69 and 60–69 years. In the subgroup individuals aged ≥ 80 years, the components of sarcopenia were all comparable among the three glucose status groups.

### Association between sarcopenia and glucose status

Multivariate logistic analyses were performed to assess the association between sarcopenia and the different glucose status, as shown in Table 3. We observed that individuals with diabetes, but not prediabetes, exhibited a significantly higher prevalence of sarcopenia compared to those with NGR, after adjusting for three different models (model 3: OR = 2.031, 95% CI 0.962–4.287 for prediabetes; OR = 3.111, 95% CI 1.630–5.940 for diabetes). When stratifying by sex, this association remained consistent in men after adjusting for multiple confounders (model 3: OR = 2.602, 95% CI 0.871–7.773 for prediabetes; OR = 5.470, 95% CI 1.551–19.296 for diabetes). However, prediabetes or diabetes did not show a significant association with sarcopenia in women after adjusting for model 3 (OR = 1.454, 95% CI 0.500–4.224 for prediabetes; OR = 1.748, 95% CI 0.790–3.868 for diabetes). Furthermore, we found that men with diabetes were significantly associated with a higher prevalence of LMS (OR = 2.616, 95% CI 1.518–4.511) and there was a marginal association with an increased rate of severe sarcopenia (OR = 10.693, 95% CI 0.955–119.73) and LMM (OR = 2.300, 95% CI 1.000–5.290) after fully adjustment of covariates, whereas women with diabetes were not significantly associated with these parameters. Additionally, prediabetes was independently associated with severe sarcopenia (OR = 3.647, 95% CI 1.532–8.697), LMS (OR = 1.472, 95% CI 1.018–2.127) and LGS (OR = 1.673, 95% CI 1.054–2.655) in the entire cohort. When stratifying by gender, we further observed that prediabetes was significantly associated with LMS in men (OR = 1.897, 95% CI 1.019–3.543) and related to LMM (OR = 3.174, 95% CI 1.287–7.829) and LGS (OR = 2.075, 95% CI 1.155–3.727) in women.

**Table 3:** Association between glucose status and the prevalence of sarcopenia, severe sarcopenia and components of sarcopenia

	NGR	Prediabetes OR (95% CI), *P*	Diabetes OR (95% CI), *P*
Sarcopenia			
Total			
Model 1	Reference	1.801 (1.102, 2.942), 0.021	2.089 (1.422, 3.068), < 0.001
Model 2	Reference	1.371 (0.857, 2.193), 0.180	1.453 (0.951, 2.222), 0.082
Model 3	Reference	2.031 (0.962, 4.287), 0.061	3.111 (1.630, 5.940), 0.002
Male			
Model 1	Reference	2.926 (1.529, 5.602), 0.002	3.681 (2.004, 6.762), < 0.001
Model 2	Reference	2.317 (1.193, 4.501), 0.015	2.758 (1.339, 5.679), 0.008
Model 3	Reference	2.602 (0.871, 7.773), 0.082	5.470 (1.551, 19.296), 0.012
Female			
Model 1	Reference	1.253 (0.693, 2.265), 0.443	1.306 (0.676, 2.526), 0.414
Model 2	Reference	0.913 (0.520, 1.600), 0.741	0.852 (0.423, 1.714), 0.642
Model 3	Reference	1.454 (0.500, 4.224), 0.466	1.748 (0.790, 3.868), 0.155
Severe sarcopenia			
Total			
Model 1	Reference	1.527 (0.661, 3.530), 0.310	2.534 (1.358, 4.730), 0.005
Model 2	Reference	1.100 (0.488, 2.482), 0.812	1.623 (0.857, 3.074), 0.132
Model 3	Reference	3.647 (1.532, 8.679), 0.006	2.844 (0.860, 9.400), 0.082
Male			
Model 1	Reference	2.002 (0.678, 5.915), 0.200	3.841 (1.539, 9.585), 0.005
Model 2	Reference	1.417 (0.451, 4.447), 0.538	2.447 (0.860, 6.963), 0.091
Model 3	Reference	6.596 (0.995, 43.723), 0.051	10.693 (0.955, 119.73), 0.054
Female			
Model 1	Reference	1.318 (0.496, 3.501), 0.568	1.879 (0.730, 4.835), 0.183
Model 2	Reference	0.943 (0.369, 2.406), 0.898	1.180 (0.445, 3.126), 0.731
Model 3	Reference	2.086 (0.526, 8.278), 0.273	0.833 (0.106, 6.541), 0.852
Low muscle strength			
Total			
Model 1	Reference	1.214 (0.923, 1.597), 0.158	1.147 (0.935, 1.406), 0.180
Model 2	Reference	1.225 (0.862, 1.742), 0.248	1.244 (0.978, 1.582), 0.073
Model 3	Reference	1.472 (1.018, 2.127), 0.041	1.502 (0.943, 2.394), 0.082
Male			
Model 1	Reference	1.905 (1.219, 2.977), 0.006	1.752(1.234, 2.487), 0.003
Model 2	Reference	1.759 (1.115, 2.773), 0.017	1.852 (1.245, 2.754), 0.004
Model 3	Reference	1.897 (1.019, 3.543), 0.044	2.616 (1.518, 4.511), 0.002
Female			
Model 1	Reference	0.981 (0.684, 1.406), 0.914	0.944 (0.704, 1.267), 0.691
Model 2	Reference	0.960 (0.615, 1.499), 0.853	0.974 (0.728, 1.303), 0.853
Model 3	Reference	1.224 (0.763, 1.962), 0.376	0.949 (0.507, 1.773), 0.859
Low muscle mass			
Total			
Model 1	Reference	1.832 (1.347, 2.491), < 0.001	2.499 (1.917, 3.258), < 0.001
Model 2	Reference	1.310 (0.963, 1.782), 0.084	1.459 (1.060, 2.010), 0.022
Model 3	Reference	1.338 (0.736, 2.434), 0.316	2.141 (1.162, 3.946), 0.018
Male			
Model 1	Reference	1.707 (1.211, 2.406), 0.003	2.669 (1.921, 3.708), < 0.001
Model 2	Reference	1.303 (0.866, 1.960), 0.195	1.649 (1.116, 2.437), 0.014
Model 3	Reference	0.570 (0.271, 1.198), 0.127	2.300 (1.000, 5.290), 0.050
Female			
Model 1	Reference	1.930 (1.328, 2.803), 0.001	2.260 (1.514, 3.372), < 0.001
Model 2	Reference	1.345 (0.963, 1.878), 0.080	1.276 (0.783, 2.080), 0.316
Model 3	Reference	3.174 (1.287, 7.829), 0.016	2.104 (0.718, 6.170), 0.161
Low gait speed			
Total			
Model 1	Reference	1.817 (1.457, 2.267), < 0.001	3.367 (2.660, 4.262), < 0.001
Model 2	Reference	1.353 (1.038, 1.763), 0.027	2.438 (1.908, 3.116), < 0.001
Model 3	Reference	1.673 (1.054, 2.655), 0.031	1.933 (1.237, 3.022), 0.007
Male			
Model 1	Reference	1.639 (1.140, 2.355), 0.009	3.446 (2.459, 4.829), < 0.001
Model 2	Reference	1.140 (0.762, 1.705), 0.511	2.468 (1.678, 3.629), < 0.001
Model 3	Reference	1.087 (0.506, 2.334), 0.819	2.325 (0.849, 6.363), 0.094
Female			
Model 1	Reference	2.090 (1.536, 2.844), < 0.001	3.630 (2.670, 4.936), < 0.001
Model 2	Reference	1.529 (1.085, 2.154), 0.017	2.432 (1.761, 3.357), < 0.001
Model 3	Reference	2.075 (1.155, 3.727), 0.018	1.482 (0.746, 2.945), 0.241

Model 1 was unadjusted

Model 2 was adjusted for age, gender, race and BMI (Gender were not adjusted in the subgroup analysis)

Model 3 was adjusted for age, gender, race, BMI, waist circumference, percent of total fat, education level, marital status, PIR, smoked at least 100 cigarettes in life, vigorous activities, sedentary activities, serum 25(OH)D, total bilirubin, uric acid, creatinine, cholesterol, history of cancer and history of osteoporosis or brittle bones (Gender were not adjusted in the subgroup analysis)

### Association between sarcopenia and HbA1c

After adjusting for multiple factors, a significant positive association was observed between HbA1c and sarcopenia in the entire cohort (OR = 1.506, 95% CI 1.149–1.974) as well as in the male group (OR = 1.993, 95% CI 1.511–2.629), but not in the female group (OR = 0.940, 95% CI 0.605–1.461) (Table 4).

**Table 4:** Association between glycohemoglobin and the prevalence of sarcopenia

	Model 1 OR (95% CI) *P-*value	Model 2 OR (95% CI) *P-*value	Model 3 OR (95% CI) *P-*value
Total	1.193 (1.071, 1.328), 0.002	1.162 (0.982, 1.375), 0.078	1.506 (1.149, 1.974), 0.006
Male	1.391 (1.197, 1.617), 0.000	1.478 (1.143, 1.912), 0.004	1.993 (1.511, 2.629), < 0.001
Female	0.992 (0.848, 1.160), 0.917	0.877 (0.684, 1.125), 0.290	0.940 (0.605, 1.461), 0.770

Model 1 was unadjusted

Model 2 was adjusted for age, gender, race and BMI (Gender were not adjusted in the subgroup analysis)

Model 3 was adjusted for age, gender, race, BMI, waist circumference, total percent fat, education level, marital status, PIR, smoked at least 100 cigarettes in life, vigorous activities, sedentary activities, serum 25(OH)D, total bilirubin, uric acid, creatinine, cholesterol, history of cancer and history of osteoporosis or brittle bones (Gender were not adjusted in the subgroup analysis)

### Assessment of public health impact of glucose status on population

PAFs for population counterfactuals were reported in Table 5. In the partial prevention, the analysis demonstrated that diabetes accounted for 16.3% (95% CI 1.8–28.6%) of observed sarcopenia cases, 12.1% (95% CI 0.8–22.1%) of observed LMM cases and 9.5% (95% CI 0.4–17.8%) of observed LGS cases. In the comprehensive prevention scenario, maintaining normal glucose status in the entire population could have prevented 38.5% (95% CI 13.7–56.2%) of sarcopenia cases, 50.9% (95% CI 21.9–69.1%) of severe sarcopenia cases, 15.2% (95% CI 3.7–25.2%) of LMS cases, 21.3% (95% CI 0.1–37.9%) of LMM cases and 27.3% (95% CI 8.0–42.6%) of LGS cases.

**Table 5:** Population attributable fractions (PAFs) for population counterfactuals of sarcopenia

	Scenario	PAF (%)	95% CI
Sarcopenia	Partial prevention (diabetes vs. non-diabetes)	16.3	1.8, 28.6
	Comprehensive prevention (total population to NGR)	38.5	13.7, 56.2
Severe sarcopenia	Partial prevention (from diabetes to non-diabetes)	5.2	− 36.5, 34.2
	Comprehensive prevention (total population to NGR)	50.9	21.9, 69.1
Low muscle strength	Partial prevention (from diabetes to non-diabetes)	3.4	− 3.4, 9.9
	Comprehensive prevention (total population to NGR)	15.2	3.7, 25.2
Low muscle mass	Partial prevention (from diabetes to non-diabetes)	12.1	0.8, 22.1
	Comprehensive prevention (total population to NGR)	21.3	0.1, 37.9
Low gait speed	Partial prevention (from diabetes to non-diabetes)	9.5	0.4, 17.8
	Comprehensive prevention (total population to NGR)	27.3	8.0, 42.6

Adjusted for age, gender, race, BMI, waist circumference, total percent fat, education level, marital status, PIR, smoked at least 100 cigarettes in life, vigorous activities, sedentary activities, serum 25(OH)D, total bilirubin, uric acid, creatinine, cholesterol, history of cancer and history of osteoporosis or brittle bones

## Discussion

To the best of our knowledge, this is the first study to utilize the comprehensive diagnostic criteria of sarcopenia, incorporating not only LMM but also by other parameters such as LMS and LGS, in assessing the relationship between sarcopenia and glucose status in US population. Our findings revealed that the prevalence of sarcopenia was higher among individuals with prediabetes and diabetes compared to those with NGR. After accounting for multiple confounding factors, we demonstrated that diabetes was an independent risk factor of sarcopenia and LMS compared to NGR in men, but not in women. Prediabetes was found to be independently associated with severe sarcopenia, LMS and LGS in the entire cohort. Furthermore, we have identified a correlation between hyperglycemia and sarcopenia in men. Importantly, our study highlighted the potential public health impact, indicating that a substantial number of sarcopenia and severe sarcopenia cases could have been prevented if the entire population had maintained NGR.

The prevalence of sarcopenia in individuals with diabetes varies significantly among different studies, ranging from 7 to 35% [19]. This wide variation can be attributed to various factors, including the diagnostic criteria used to evaluate sarcopenia, age range, gender, ethnicity/race and comorbidities of the participants. For instance, even when using the same diagnostic standard such as criteria established by the FNIH, some studies only define sarcopenia based on LMM [15], while others defined sarcopenia as a combination of reduced muscle mass and reduced muscle strength [20]. In our study, we adopted the latter approach to emphasize the importance of muscle strength in the diagnosis of sarcopenia. We found that the prevalence of prediabetes and diabetes in US adults aged 50 years and older was 6.6% and 7.6%, respectively.

Previous studies have investigated the association between glucose homeostasis and the prevalence of sarcopenia based on muscle mass, strength or performance [7–9]. A Chinese study [21] estimated that patients with T2D had a 1.37-fold higher likelihood of sarcopenia compared to healthy subjects in a community-dwelling citizens aged 60–95 years. Bouchi et al. [22] reported a significantly increased prevalence of sarcopenia in individuals with latent autoimmune diabetes in adults (LADA) (OR 9.57, 95% CI 1.86–49.27) and a marginally elevated prevalence in those with T2D (OR 2.99, 95% CI 0.93–10.80). In our current study, conducted with a nationally representative sample, we indicated that participants with diabetes had a 3.11-fold higher proportion of sarcopenia compared to those with NGR. Moreover, we observed a gender disparity in this relationship, whereby diabetic men were more susceptible to sarcopenia than diabetic women. This finding was consistent with a previous meta-analysis study [19] and the English Longitudinal Study of Ageing study [23]. The gender distribution of susceptibility to sarcopenia may be attributed to faster muscle degeneration in men and a gradually fat increase in women during aging, and the change pattern in estrogen, testosterone and insulin-like growth factor-1 levels [24].

Limited studies have explored the association between prediabetes and the muscle system. A study [13] conducted in an Asian Indian population recruited men aged 20–50 years and found similar skeletal muscle mass but lower muscle torque in individuals with prediabetes (n = 125) compared with healthy controls (n = 44). A Japanese study [14] involving 1629 older adults indicated that sarcopenia was associated with prediabetes in men (OR 2.081, 95% CI 1.031–4.199), but not in women (OR 2.081, 95% CI 1.031–4.199). Furthermore, several studies investigated the relationship between grip strength and prediabetes. A large-scale cohort study from China [25] comprising 27,295 participants aged 20–90 years demonstrated that a one-unit increase in grip strength per body weight would result in a 52% decrease in the risk of prediabetes for men (OR 0.48, 95% CI 0.30–0.74) and a 62% decrease for women (OR 0.38, 95% CI 0.20–0.70). Considering the influence of ethnicity on study findings and the majority of research being conducted in Asian population, the relationship between prediabetes and sarcopenia in non-Asians has not been definitively established. In our study, we provided further evidence in this area by demonstrating that prediabetes was significantly associated with severe sarcopenia, LMS and LGS in the entire cohort after adjusting for multiple factors. These findings expand our understanding of relationship between prediabetes and sarcopenia. Given that sarcopenia is a potent predictor of poor health outcomes, it is of critical significance to facilitate early intervention to prevent or delay the progression of sarcopenia.

The inverse association between hyperglycemia and the course of sarcopenia has not been previously reported in a consistent manner. A Korean study [26] suggested a HbA1c level of ≥ 8.5% in elderly men with diabetes significantly impaired leg muscle quality and physical performance. In contrast, another study [27] showed that poor glucose control, as indicated by higher HbA1c levels, mainly resulted in a decline in SMI in patients with T2D, rather than affecting muscle quality and physical performance such as grip strength and gait speed. The discrepancy in findings might be contributed to ethnic difference, study design settings and the diagnostic criteria used for sarcopenia. Our study showed a strong inverse association between chronic hyperglycemia and sarcopenia in male (OR 1.993, 95% CI 1.511–2.629), but not in female (OR 0.940, 95% CI 0.605–1.461). Chronic hyperglycemia can lead to elevated levels of advanced glycosylation end products (AGEs) and inflammatory cytokines, which may exacerbate the loss of muscle mass and strength [28, 29]. Interestingly, a post hoc analysis of our data revealed that HbA1c was significant higher in male compared to female, which may partly explain the gender difference observed in the association between hyperglycemia and sarcopenia.

PAF is a valuable statistic used to estimate the burden of a certain disease attributed to a specific risk factor [30]. By calculating the PAF, researchers and policymakers can assess the potential public health impact of interventions targeting modifiable risk factors. This information is crucial for developing effective prevention and control strategies. In our study, we applied PAFs to quantify the impact of glucose status on sarcopenia and to evaluate hypothetical scenarios. To our knowledge, this was the first study to utilize PAFs in assessing the relationship between glucose and sarcopenia. Our findings revealed that 16.3% of sarcopenia cases in the total population could be prevented if the participants with diabetes were able to change to non-diabetic state, while 38.5% of sarcopenia cases and 50.9% of severe sarcopenia case would be potentially eliminated if the entire population maintained NGR. These results emphasize the importance of preventing the progression from NGR to prediabetes or diabetes in order to mitigate the burden of sarcopenia. Targeted programs may be designed by public health policymakers to promote healthy lifestyles, diabetes prevention, and early intervention strategies at both individual and population levels.

It is widely recognized that Caucasians with T2D tend to have higher BMI compared to their Asian counterparts [31]. Moreover, studies indicated that the importance of accounting for obesity when evaluating muscle mass [17, 32]. In patients with obesity, prediabetes or diabetes, assessing muscle mass using ALM/BMI was considered more accurate than using ALM/height2 [33]. Additionally, since abdominal fat can increase without significant changes in BMI or overall weight [34], other obesity indices such as waist circumference and total fat percentage should also be taken into consideration when examining the relationship between glucose status and muscle mass. In our present analysis, we reported that compared to individuals with NGR, those with diabetes had an approximately 3.1-fold higher prevalence of sarcopenia after adjusting for multiple confounders, including waist circumference and total fat percentage.

Several underlying mechanisms are involved in the association between prediabetes/diabetes and sarcopenia. Firstly, insulin resistance has been shown to negatively affect mitochondrial function, protein anabolism and autophagy pathways in the muscle system, thereby impacting skeletal muscle mass and strength [35, 36]. Secondly, chronic low-grade inflammation plays an essential role in the development of sarcopenia [37]. Thirdly, the decline in anabolic hormone activity, including IGF-I, testosterone, ghrelin, further exacerbates the detrimental effects of diabetes on muscle health [38]. Fourth, vitamin D deficiency is prevalent in diabetic patients and studies have highlighted the role of active vitamin D in regulating genes and signaling pathways involved in muscle cell proliferation and differentiation, and improvement of muscle strength by suppressing the expression of myostatin, a negative regulator of muscle growth [39, 40]. Lastly, malnutrition or risk of malnutrition is particularly high in elderly patients with diabetes [41, 42], which significantly increases the risk of sarcopenia [43].

There were several limitations of our study. First, since NHANES is cross-sectional survey, the causal relationship between glucose status and sarcopenia could not be assessed. Second, NHANES only included the non-institutionalized US population, and it is unable to get DXA data if frail or elderly participants cannot to attend the MEC, thus may underestimating the rate of sarcopenia. Third, NHANES assessed grip strength in different survey cycles than body composition, thus precluding to evaluate grip strength in this study. Fourth, although we controlled several potential confounders, such as demographic indexes, lifestyle factors, biochemical parameters, waist circumference, percentage of total body fat, history of cancer or osteoporosis/brittle bones, the findings may be affected by other confounders such as dietary and thyroid function. Finally, given the missing data of diabetic medications in NHANSE database, types of diabetic medicine were not adjusted in our study.

## Conclusions

In summary, we revealed that prediabetes and diabetes were independently associated with the prevalence and the severity of sarcopenia in US population. A substantial proportion of sarcopenia cases could be prevented by effectively managing and slowing down the progression of hyperglycemia. Future studies with longitudinal follow-up are needed to validate the association between glucose status and sarcopenia and gain a deeper understanding of the underlying pathophysiology.

### Supplementary Information

Supplementary Material 1: Supplementary Table. The prevalence of sarcopenia in US participants with NGR, prediabetes and diabetes stratified by sex and age.

## Data Availability

The datasets of this study was extracted from NHANES website. The data that support the findings of this study are available from the corresponding author upon reasonable request.
