# Supplementary material for: Prediabetes and diabetes were attributed to the prevalence and severity of sarcopenia in middle-aged and elderly adults
Source: Diabetol Metab Syndr. 2024 Jun 2;16:122. doi: 10.1186/s13098-024-01355-3 (PMC11145839; doi:10.1186/s13098-024-01355-3)
Supplement: Supplementary file 1 — Supplementary Material 1: Supplementary Table. The prevalence of sarcopenia in US participants with NGR, prediabetes and diabetes stratified by sex and age. [file 13098_2024_1355_MOESM1_ESM.docx]

**Supplementary Table 1 The prevalence of sarcopenia in US participants with NGR, prediabetes and diabetes stratified by sex and age**

|  |  | Male  (n=1976) | | | |  | Female  (n=2026) | | | |
| --- | --- | --- | --- | --- | --- | --- | --- | --- | --- | --- |
|  |  | NGR  (n=904) | Prediabetes  (n=614) | Diabetes  (n=458) | *P* |  | NGR  (n=1035) | Prediabetes  (n=558) | Diabetes  (n=433) | *P* |
| Total | Sarcopenia (%) | **2.7(1.7-4.2)** | **7.5(5.0-11.0)** | **9.2(6.0-13.8)** | **<0.001** |  | 4.7(3.5-6.3) | 5.8(3.8-8.8) | 6.0(3.8-9.6) | 0.567 |
|  | Severe sarcopenia (%) | **1.2(0.6-2.2)** | **2.3(1.1,4.5)** | **4.3(2.3,7.9)** | **0.017** |  | 1.9(1.1-3.4) | 2.5(1.2-5.3) | 3.6(1.9-6.5) | 0.401 |
|  | Low muscle strength(%) | **13.1(10.3-16.4)** | **22.3(17.6-27.8)** | **20.9(16.1-26.6)** | **0.004** |  | 28.7(25.4-32.3) | 28.3(22.5-35.0) | 27.6(21.7-34.3) | 0.928 |
|  | Low muscle mass(%) | **13.2(11.0-15.7)** | **20.1(16.2-25.7)** | **28.8(23.5-34.8)** | **<0.001** |  | **10.8(8.7-13.3)** | **19.0(14.0-25.2)** | **21.5(17.0-26.8)** | **<0.001** |
|  | Low gait speed(%) | **8.2(6.4-10.5)** | **12.8(10.1-16.0)** | **23.6(19.0-28.8)** | **<0.001** |  | **14.3(11.7-17.4)** | **25.8(21.5-30.8)** | **37.7(32.7-42.9)** | **<0.001** |
| 50-59 years | Sarcopenia (%) | **0.1(0.0-0.5)** | **1.9(0.5-7.8)** | **5.0(1.4-16.5)** | **0.006** |  | 3.0(1.7-5.1) | 3.6(1.2-10.4) | 3.0(0.9-10.2) | 0.923 |
|  | Severe sarcopenia (%) | 0 | 0 | 0.4(0.0-2.8) | 0.344 |  | 1.1(0.3-3.6) | 0.9(0.2-4.8) | 1.1(0.3-3.5) | 0.967 |
|  | Low muscle strength(%) | 6.7(4.3-10.4) | 11.5(6.1-20.4) | 15.5(7.4-29.6) | 0.102 |  | 15.5(12.8-18.6) | 19.6(12.8-28.8) | 19.3(11.0-31.5) | 0.464 |
|  | Low muscle mass(%) | **6.1(4.1-9.1)** | **12.4(7.3-20.3)** | **19.1(9.5-34.6)** | **0.014** |  | **7.2(4.9-10.5)** | **10.6(5.3-20.1)** | **19.7(11.0-32.7)** | **0.028** |
|  | Low gait speed(%) | **3.8(2.2-6.5)** | **3.3(1.4-7.7)** | **15.0(8.3-25.6)** | **0.001** |  | **4.5(2.5-7.8)** | **11.6(7.6-17.5)** | **23.7(14.5-36.3)** | **<0.001** |
| 60-69 years | Sarcopenia (%) | 3.2(1.2-8.1) | 4.9(2.1-11.4) | 11.3(6.0-20.3) | 0.056 |  | 2.8(1.2-6.0) | 4.6(1.8-11.3) | 4.4(1.7-10.9) | 0.620 |
|  | Severe sarcopenia (%) | **1.9(0.6-5.4)** | **1.2(0.2-6.7)** | **7.2(3.0-16.1)** | **0.049** |  | 0.6(0.1-2.6) | 2.5(0.7-9.0) | 3.7(1.2-10.7) | 0.102 |
|  | Low muscle strength(%) | 14.2(9.3-21.0) | 20.1(13.1-29.7) | 19.4(12.5-28.8) | 0.416 |  | 33.4(26.9-40.5) | 26.7(18.7-36.6) | 28.4(19.9-38.8) | 0.376 |
|  | Low muscle mass(%) | **14.8(11.0-19.6)** | **16.7(11.1-24.4)** | **32.9(24.1-43.0)** | **0.001** |  | **10.1(6.8-14.8)** | **20.6(13.2-30.7)** | **19.7(12.8-28.9)** | **0.023** |
|  | Low gait speed(%) | **4.6(2.6-8.1)** | **7.0(4.1-11.9)** | **20.7(13.7-29.9)** | **<0.001** |  | **11.6(8.7-15.3)** | **22.7(16.1-31.2)** | **39.5(31.1-48.6)** | **<0.001** |
| 70-79 years | Sarcopenia (%) | 8.0(4.1-15.0) | 19.0(10.5-31.8) | 9.2(5.1-15.9) | 0.053 |  | 7.7(4.6-12.6) | 10.0(6.4-15.2) | 8.5(3.4-19.7) | 0.778 |
|  | Severe sarcopenia (%) | 2.5(1.0-6.1) | 6.4(2.7-14.2) | 4.2(1.4-12.0) | 0.232 |  | 2.5(0.8-7.4) | 4.4(1.5-12.4) | 4.3(1.4-12.6) | 0.675 |
|  | Low muscle strength(%) | 25.9(19.6-33.5) | 40.3(28.8-53.0) | 26.7(17.3-38.8) | 0.109 |  | 48.6(41.6-55.7) | 36.7(25.8-49.1) | 31.3(20.1-45.3) | 0.076 |
|  | Low muscle mass(%) | 29.6(22.1-38.2) | 34.7(26.7-43.6) | 34.1(25.4-44.1) | 0.538 |  | 15.5(11.1-21.2) | 26.4(17.2-38.3) | 24.6(15.9-35.9) | 0.062 |
|  | Low gait speed(%) | **18.6(13.6-25.0)** | **24.3(18.6-31.1)** | **37.5(28.0-48.2)** | **0.002** |  | **22.5(15.5-31.5)** | **33.6(22.8-46.4)** | **44.1(32.1-56.8)** | **0.019** |
| ≥80 years | Sarcopenia (%) | 12.0(6.0-22.6) | 16.4(12.4-21.3) | 23.2(12.3-39.4) | 0.251 |  | 14.8(8.5-24.7) | 7.1(3.3-14.8) | 13.3(6.8-24.5) | 0.274 |
|  | Severe sarcopenia (%) | 6.3(2.2-17.0) | 7.5(4.0-13.7) | 10.1(4.3-21.7) | 0.708 |  | 10.1(4.7-20.4) | 4.0(1.5-10.2) | 8.2(3.4-18.6) | 0.320 |
|  | Low muscle strength(%) | 38.3(28.2-49.5) | 40.9(34.0-48.2) | 46.8(33.1-61.0) | 0.517 |  | 49.2(39.4-59.1) | 42.5(30.2-55.9) | 39.0(22.6-58.3) | 0.510 |
|  | Low muscle mass(%) | 31.2(21.2-43.3) | 41.1(31.8-51.0) | 44.3(29.5-60.3) | 0.207 |  | 24.1(15.8-35.0) | 26.0(15.4-40.6) | 25.5(15.7-38.7) | 0.903 |
|  | Low gait speed(%) | 43.7(32.4-55.6) | 51.1(41.6-60.6) | 48.9(32.8-65.1) | 0.530 |  | 61.8(49.0-73.1) | 61.4(50.2-71.5) | 55.8(40.7-70.0) | 0.772 |

Notes: proportion for categorical variables.

Abbreviation: NGR: normal glucose regulation
